# The positive effects of Mn^2+^ on nitrogen use and surfactin production by *Bacillus subtilis* ATCC 21332

**DOI:** 10.1080/13102818.2015.1006905

**Published:** 2015-02-12

**Authors:** Xiangfeng Huang, Jia'nan Liu, Yihan Wang, Jia Liu, Lijun Lu

**Affiliations:** ^a^ College of Environmental Science and Engineering, State Key Laboratory of Pollution Control and Resource Reuse, Key Laboratory of Yangtze River Water Environment of the Ministry of Education, the Collaborative Innovation Center of Advanced Technology and Equipment for Water Pollution Control, the Collaborative Innovation Center for Regional Environmental Quality, Tongji University, Shanghai, China

**Keywords:** *Bacillus subtilis*, surfactin, Mn^2+^, enzyme activity, nitrogen metabolism

## Abstract

Surfactin, one of the most effective biosurfactants, has great potential in commercial applications. Studies on effective methods to reduce surfactin’s production cost are always a hotspot in the research field of biosurfactants. The aim of this study was to reveal the role of Mn^2+^ in promoting the biosynthesis of surfactin by *Bacillus subtilis* ATCC 21332, which could arise more targeted suggestions on surfactin yield promotion. In this study, *B.* *subtilis* was cultivated in media containing different Mn^2+^ concentrations. The obtained results showed that the yield of surfactin gradually increased upon Mn^2+^ addition (0.001 to 0.1 mmol/L) and achieved the maximal production of 1500 mg/L, which reached 6.2-fold of the yield obtained in media without Mn^2+^ addition. Correspondingly, the usage ratios of ammonium nitrate were improved. When the Mn^2+^ concentration was higher than 0.05 mmol/L, nitrate became the main nitrogen source, instead of ammonium, indicating that the nitrogen utilization pattern was also changed. An increase in nitrate reductase activity was observed and the increase upon Mn^2+^ dosage had a positive correlate with nitrate use, and then stimulated secondary metabolic activity and surfactin synthesis. On the other hand, Mn^2+^ enhanced the glutamate synthase activity, which increased nitrogen absorption and transformation and provided more free amino acids for surfactin synthesis.

## Introduction

Biosurfactants are surface-active compounds, produced by a variety of micro-organisms.[1–3] Because of their high biodegradability, low toxicity and high efficiency, biosurfactants are of increasing interest as possible alternative to chemical surfactants.[4,5] Based on their chemical composition, biosurfactants can be categorized into five groups, which include glycolipids, lipopeptides, phospholipids, fatty acids and polymeric biosurfactants.[6–8] Surfactin, which belongs to lipopeptides group, produced by *Bacillus subtilis*, is recognized as one of the most effective biosurfactants.[9] It consists of a heptapeptide head group attached to a lactone ring by a beta-hydroxy fatty acid.[10] As it can reduce the surface tension to 27 mN/m at concentration as low as 0.005%,[9,11–14] surfactin has a great potential in commercial applications.[15] However, its high production costs and low yield limit its commercial use. Medium improvement is one of the most common and effective approaches to promote surfactin production.[9,16]

Methods for medium improvement mainly involve optimization of the carbon source,[17–19] nitrogen source [20–23] and promoting factors. This includes selection of nutrient species and optimization of their concentrations and dosing. As one of the most important promoting factors, metal ions have attracted attention for their ability to enhance surfactin yields. Of the most commonly investigated metal ions, which are Mn^2+^, Cu^2+^, Co^2+^, Mg^2+^, Ni^2+^, Fe^2+^, Ca^2+^, Al^3+^ and Zn^2+^, Mn^2+^ has shown to be one of the metal ions that can promote surfactin yield significantly.[24–27] Studies have shown that the addition of Mn^2+^ shortened the cycle duration in continuous-phased growth of *B. subtilis* [28] and increased its surfactin productivity in batch cultures.[27] Wei and Chu speculated that changes in nitrogen usage and K^+^ uptake were the reasons for the promotion of surfactin synthesis by Mn^2+^.[27] However, these studies only focused on changes in the surfactin yield, but none of them studied the mechanisms which are directly responsible for the influence of Mn^2+^ on surfactin yield enhancement.

Sheppard and Cooper reported an intimate relationship between the availability of Fe^2+^ and Mn^2+^ and the usage of nitrogen.[28] The essential role of amino acids as precursors of the heptapeptide head group of surfactin makes nitrogen metabolism very important for the surfactin biosynthesis by *B. subtilis*. Davis et al. found that when *B. subtilis* was cultivated in a defined medium with ammonium nitrate as the nitrogen source, the surfactin production was highest when *B. subtilis* used nitrate, causing the subsequent onset of nitrate-limited growth.[29] Mn^2+^ is the most important trace metal element for the growth of some species of *Bacillus* and fungi, and it can function as a cofactor for many enzymes involved in nitrogen metabolism.[30] When *B. subtilis* is cultivated in media with ammonium nitrate as the nitrogen source, nitrogen assimilation includes the usage of NH_4_^+^ and NO_3_^−^. Before the assimilation of NO_3_^−^, it must be transformed into NO_2_^−^ and then into NH_4_^+^.[31] This process is limited by the first step reaction, which is catalyzed by nitrate reductase (NR).[32] For *B. subtilis*, assimilation and transformation of NH_4_^+^ into an organic form primarily occurs through the coupled reactions catalyzed by glutamine synthetase (GS) and glutamate synthase (GOGAT)[33]

(1) $$\text{GS:}\;{\text{NH}}_{3}+\text{glutamate}+\text{ATP}\overset{{\text{Mn}}^{2+}\;\text{or}\;{\text{Mg}}^{2+}}{⟶}\text{glutamine}+\text{ADP}+\text{Pi,}$$

(2) $$\text{GOGAT}:\text{glutamine}+\alpha -\text{ketoglutarate}+\text{NADPH}\to 2\;\text{glutamate}+{\text{NADP}}^{+}.$$

This process is the only pathway for glutamine and glutamate synthesis and ammonium assimilation in *B. subtilis*’ cells.[34] Thus, the three enzymes are crucial to nitrogen metabolism, growth and reproduction of *B. subtilis.*

In this study, we cultivated *B. subtilis* in media with different initial Mn^2+^ concentrations and then monitored the growth characteristics, surfactin production, carbon and nitrogen usage characteristics and activities of the enzymes, which are involved in nitrogen assimilation. Taken together, we hoped that the results would help to reveal the mechanism that is responsible for the influence of Mn^2+^ on the biosynthesis of surfactin by *B. subtilis*.

## Materials and methods

### Micro-organisms and culture conditions

*B.* *subtilis* ATCC 21332 was obtained from the American Type Culture Collection. The strain was maintained on an agar slant at 4 °C and transferred monthly.

*B.* *subtilis* ATCC 21332 was cultured in agar plate at 30 °C for two days, then inoculated into 100 mL nutrient broth in 250-mL flasks and incubated on a gyratory shaker at 200 rpm for 12–14 h at 30 °C. For the preparation of the next seed culture, 5 mL of the cultured medium were inoculated into 100 mL of fresh medium. This seed culture was incubated for 10 h at 200 rpm at 30 °C before inoculating 1 mL from it into a 500-mL flask, which contained 200 mL of fermentation medium with different concentrations of Mn^2+^. The fermentation medium flasks were incubated under the same conditions as the seed culture, for six days. The composition of the three media was as follows: the nutrient broth contained 10 g/L NaCl, 5 g/L yeast extract and 10 g/L fish peptone; the seed and fermentation media were based on the mineral salt medium reported by Cooper et al.[26] The seed medium contained 40 g/L glucose, 50 mmol/L NH_4_NO_3_, 30 mmol/L Na_2_HPO_4_, 30 mmol/L KH_2_PO_4_, 7 μmol/L CaCl_2_ and 4 μmol/L ethylene diamine tetraacetic acid disodium (Na-EDTA). The composition of the fermentation medium contained all the components of the seed medium with three additional components, including 0.8 mmol/L MgSO_4_, 4 μmol/L FeSO_4_ and a certain concentration of MnSO_4_ (0, 0.001, 0.005, 0.05 and 0.1 mmol/L, respectively).

### Analysis of enzyme activities

#### Preparation of cell extracts

After centrifugation of culture samples (19800 × *g*, 10 min, 4 °C), the cell pellets were washed twice with distilled water, once with phosphate buffer (pH 7.7) and then re-suspended in buffer to remove surfactant that would interfere with the subsequent enzyme analyses. The cell suspensions were mixed with glass beads (diameter 0.1 mm) and disrupted using the bead-milling method (3000 rpm, 5 min). Cell debris was removed by centrifugation (13750 × *g*, 15 min, 4 °C). The supernatants were stored at 30 °C in a water bath and used as crude enzyme for enzyme assays.

#### Analysis of glutamine synthetase

The biosynthetic activity of GS was measured at 30 °C by detecting the production of a red complex produced by the interaction of Fe^3+^ with *γ*-glutamylhydroxamate, as described by Shapiro and Stadtman.[35] The GS activity was expressed in terms of the absorbance of the solution at 540 nm. The control group had buffer instead of crude enzyme in the reaction.

#### Analysis of nitrate reductase and glutamate synthase

NR and GOGAT were assayed spectrophotometrically by measuring reduced nicotinamide adenine dinucleotide (NADH) oxidation at 340 nm (Δ*OD*/min) as described by Berges and Harrison [36] and Meers et al.,[37] respectively. For this purpose, 0.6 mL of crude enzyme was added into 2.4 mL of the reaction system A, which contained 80.2 mmol/L K_2_HPO_4_, 19.8 mmol/L KH_2_PO_4_, 100 mmol/L KNO_3_ and 0.2 mmol/L NADH and maintained for 3 min to determine the NR activity. Next, 0.6 mL of crude enzyme was added into 2.4 mL of the reaction system B, which contained 20 mmol/L Tris-HCl (pH 7.0), 1 mmol/L *α*-ketoglutaric acid, 15 mmol/L L-glutamine and 0.1 mmol/L NADH and maintained for 3 min to determine the GOGAT activity. The temperature of the spectrophotometer (UV-8000; Shanghai Yuanxi Instruments Ltd., Shanghai, China) was maintained at 30 °C during the assays. The concentration of protein was detected using Coomassie brilliant blue. The NR and GOGAT activities were obtained from the following equation:

(3) $$\text{Enzyme}\;\text{specific activity}(U/\text{mg})=\frac{\left(\frac{\Delta OD}{\min}\right)\times Vt\times DF}{6.22\times 1.0\times Vs\times C}.$$

Equation (3) takes into account the NADH oxidation at 340 nm (Δ*OD*/min), the total reaction volume (*Vt*, mL), the crude enzyme volume (*Vs*, mL), the dilution factor (*DF*) and the protein concentration of crude enzyme (*C*, mg/L).

### Analysis methods

#### Determination of biomass

Samples were centrifuged (19800 × *g*, 4 °C) for 10 min. The pellet was suspended in distilled water and re-centrifuged. The biomass was determined by weighing after drying by vacuum lyophilization at −50 °C for 24 h.

#### Quantitative analysis of surfactin

The concentration of surfactin was determined by using a reverse-phase, high-performance liquid chromatograph (HPLC), (Waters Alliance2695-2489, Waters, USA) equipped with a C18-WR column (5 μm, 250 × 4.6 mm). To determine the concentration of surfactin, culture samples were withdrawn aseptically and centrifuged at 13,750 × *g* for 10 min to pellet the cells. The supernatant was dried by vacuum lyophilization at −50 °C for 24 h and re-dissolved in methanol in order to avoid contamination by saline ions. The methanol solution of surfactin was filtered through a 0.45-μm membrane and quantified by HPLC. The mobile phase was 0.05% trifluoroacetic acid/acetonitrile (1/9, v/v) at 0.5 mL/min with a column temperature of 35 °C and a sample size of 20 μL. The absorbance of the eluent was monitored at 205 nm. Surfactin purchased from Sigma (St. Louis, MO, USA) served as a standard.

#### Determination of glucose, nitrite, nitrate and ammonium

Culture samples were centrifuged (19,800 × *g*, 4 °C) for 10 min to pellet the cells. The supernatant was used for direct glucose determination. In order to determine the concentration of nitrite, nitrate and ammonium, the supernatant was acidified to pH < 2.0 with sulphuric acid (10%, v/v) and centrifuged at 19,800 × *g* for 10 min to remove the precipitated surfactant.

Glucose was determined by using 3,5-dinitrosalicylic acid. The content of nitrate was determined by UV spectrophotometry. The concentration of nitrite was determined by using *N*-(1-naphthyl)-ethylenediamine spectrophotometry. The concentration of ammonium was determined by H_2_SO_4_ titration after distillation on a Kjeldahl determination device (K9840; Hanon Instruments Ltd, Nanjing, China). Instrument parameters were 50 mL boric acid (2%), 10 mL sodium hydroxide (40%), 6-min distillation time and 40-mL water wash.

## Results and discussion

### Biomass and surfactin production in media with different concentrations of Mn^2+^

**Figure 1.**
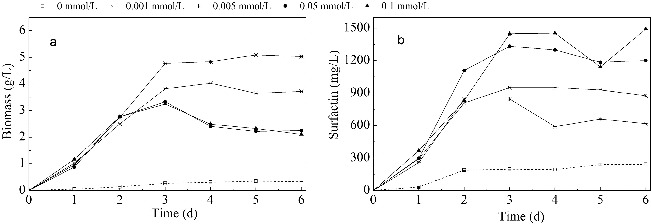
Biomass and surfactin production in media with different concentrations of Mn^2+^. Note: Biomass concentration (a); surfactin concentration (b**)**.

The biomass and surfactin concentrations during the cultivation are shown in Figure 1. Improved cell growth and increased surfactin production were observed in media with included Mn^2+^. Initially, the biomass concentration increased before its increment when the Mn^2+^ concentration became above 0.005 mmol/L. The highest biomass was obtained when the concentration of Mn^2+^ was 0.005 mmol/L, which was 11.4 times more than that of the control group and with 50% more than the biomass in media with higher concentrations of Mn^2+^ (0.05 and 0.1 mmol/L). Thus, media containing lower concentrations (0.001 and 0.005 mmol/L) of Mn^2+^ were better for *B. subtilis* cell growth. With the increase of Mn^2+^ dosage, the surfactin concentration was enhanced. The highest yield was 1500 mg/L when the concentration of Mn^2+^ was 0.1 mmol/L, which was 6.2 times more than that of the control group and 1.5 times more than that of the groups with lower Mn^2+^ concentrations (0.001 and 0.005 mmol/L). Therefore, it can be concluded that the higher concentrations of Mn^2+^ (0.05 and 0.1 mmol/L) were better for the production of surfactin from *B. subtilis* strain.

It is clear that the addition of Mn^2+^ can significantly improve the growth of *B. subtilis* and the production of surfactin. In the study of Wei and Chu,[27] the biomass and surfactin yield were increased by 3-folds and 6.9-folds, respectively, with 0.01 mmol/L Mn^2+^ concentration. In this study, the two parameters were increased by 7-folds and 5.2-folds, respectively, with a Mn^2+^ concentration of 0.1 mmol/L. The production of surfactin by *B. subtilis* has been observed to be closely related to cell growth.[38] However, different trends of cell growth and surfactin production have been observed when Mn^2+^ concentration increased. As described by Sheppard and Cooper,[28] the cycle duration and CMC^−1^ (dilution factor of the free-cell fermentation broth that corresponds to the critical micelle concentration [26]) of the fermentation broth changed from 90 min and 7 to 180 min and 8, respectively, when the Mn^2+^ concentration increased from 0.03 to 0.3 mmol/L, which indicated that with the increase of the Mn^2+^ concentration, the rate of growth decreased by half, but the yield of surfactin increased slightly. In this study, when the Mn^2+^ concentration was above 0.005 mmol/L (including 0.005 mmol/L), the biomass concentration decreased, while the surfactin concentration increased. Similar results have also been reported in studies, where other metal ions like Ca^2+^, Fe^2+^, K^+^ and Mg^2+^ were added as promoting factors.[24,39] One of the possible reasons may be that some enzymes have two metal binding sites which have different affinity to the metal ions. At different concentrations, alterations in the configuration and activity of the enzyme (protein) may be observed, depending on whether the binding sites are occupied.[40] More changes in exact metabolic processes and enzymatic activities that related to surfactin synthesis, due to the addition of different concentrations of Mn^2+^, will be discussed in details below.

### Usage of glucose and ammonium nitrate in media with different concentrations of Mn^2+^

#### Usage of glucose

**Figure 2.**
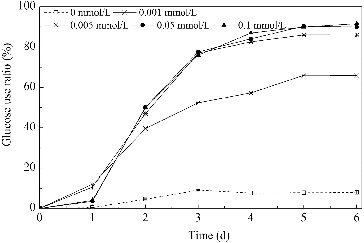
Usage of glucose in media with different concentrations of Mn^2+^.

In this study, *B. subtilis* was cultivated in media with glucose as the carbon source at an initial concentration of 40 g/L. The usage ratio of glucose, under different concentrations of Mn^2+^, is shown in Figure 2. Nutrition absorption, cell growth and surfactin production took place during the first three days of the cultivation period. The addition of Mn^2+^ promoted the usage of glucose significantly. When the Mn^2+^ concentration was 0.001 mmol/L, the usage ratio was 66%, which was nine times more than that of the control, but lower than the other three groups. At the other three concentrations of Mn^2+^, the glucose usage ratio was essentially constant -- at about 85%, reaching 11.6 times more than that of the control group. These results indicated that the glucose usage of *B. subtilis* improved significantly in the presence of low Mn^2+^ concentrations, but failed to further improve when the Mn^2+^ concentration was higher than 0.005 mmol/L.

#### Usage of ammonium nitrate

**Figure 3.**
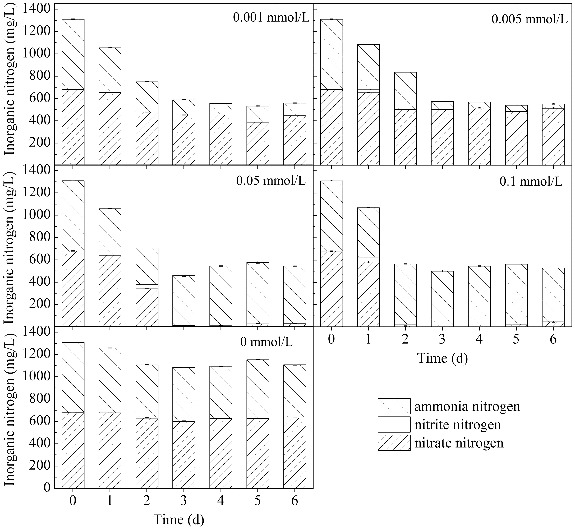
Changes in the concentration of ammonium nitrate in media with different concentrations of Mn^2+^.

Ammonium nitrate was used as the nitrogen source in this study. The use of ammonium nitrate under the influence of different concentrations of Mn^2+^ is shown in Figure 3. The usage ratio of the total inorganic nitrogen was about 60% in the media with Mn^2+^ (nitrogen concentration was stabilized at around 540 mg/L) compared with 16% for the control. Similar to the glucose use ratios, very little difference was observed between the four nitrogen usage ratios for the media with different Mn^2+^ concentrations. When the Mn^2+^ concentration was 0.005, 0.05 and 0.1 mmol/L, the surfactin yield rose gradually while the usage of carbon and nitrogen remained relatively stable. This phenomenon indicated that a bigger amount of the carbon and nitrogen nutrition, assimilated by *B. subtilis*, goes for surfactin synthesis as the Mn^2+^ concentration increased.

Ammonium nitrate consists of NH_4_^+^ and NO_3_^−^, both of which can be assimilated by *B. subtilis*. As shown in Figure 3, the usage of NH_4_^+^ and NO_3_^−^ is totally different, depending on the concentration of Mn^2+^ (0.001–0.1 mmol/L) in the medium, although the usage ratio of ammonium nitrate was similar among the different conditions. For groups with lower Mn^2+^ concentrations (0.001 and 0.005 mmol/L), the NH_4_^+^ usage of *B. subtilis* improved from 25% of the control group to 93%, while the NO_3_^−^ usage ratio only increased from 12% to 34%. Thus, NH_4_^+^ was found to be the main nitrogen source adsorbed by *B. subtilis* from these media. For groups grown in the presence of higher Mn^2+^ concentrations (0.05 and 0.1 mmol/L), the NO_3_^−^ usage ratio increased and reached 100% in three days after the start of the cultivation and as a result, the primary form of nitrogen, assimilated by *B. subtilis*, was shifted to NO_3_^−^. The decline of NH_4_^+^ was much slower, and even had a tendency to slightly increase after a two-day cultivation. In addition, during the usage of nitrate, tens of milligrams of nitrite per litre were detected. An increase of about 100 mg/L in ammonium were also detected in media with 0.05 and 0.1 mmol/L Mn^2+^ from two to four and from one to two days, respectively.

Micro-organisms’ nitrogen utilization is dramatically influenced by metal ions in the culture medium. Kim et al. [41] reported that the isolated *Kle* *b* *siella pneumoniae* F-5-2 can utilize 100% NH_4_^+^ and 30% NO_3_^−^ in 24 h. When 0.1 mmol/L Fe^2+^ was added to the medium, both NH_4_^+^ and NO_3_^−^ were exhausted simultaneously in the culture within six hours of incubation. Zhou et al. [42] found that Zn^2+^, Mn^2+^, Fe^2+^ and Fe^3+^ markedly improved the usage of nitrogen source by *Pseudomonas aeruginosa* NBRC 12389. What’s more, Zn^2+^ and Mn^2+^ only improved the utilization ratio of NH_4_^+^ (from 35% to 90%), while Fe^2+^ and Fe^3+^ improved the utilization ratios of NH_4_^+^ and NO_3_^−^, simultaneously (from 35% and 5% to 98% and 100%, respectively). Compared with the study of Sheppard and Cooper,[28] the present research focused on the influence of Mn^2+^ on the utilization of ammonium and nitrate, simultaneously. The results showed that with the addition of Mn^2+^, the usage of nitrogen source increased significantly by *B. subtilis*. The utilization of NO_3_^−^ was increased when the dosage of Mn^2+^ increased and it turned to the main nitrogen source instead of NH_4_^+^ in the cultivation of *B. subtilis*. The mechanism of the influence of metal ions on nitrogen use has not been revealed by now. One possible influence pathway is that the metal ions, added into the medium, stimulated and changed the activities of enzymes in the nitrogen metabolism process. Thus, we studied the relationship between Mn^2+^ and the key enzymes in nitrogen metabolism in the following.

In addition, as shown in Figure 1, a higher NH_4_^+^ usage ratio and biomass concentration were obtained in media with lower Mn^2+^ concentrations (0.001 and 0.005 mmol/L), while a higher NO_3_^−^ use ratio and higher surfactin production were obtained in media with higher Mn^2+^ concentrations (0.05 and 0.1 mmol/L). These results indicated that the use of NH_4_^+^ was better for *B. subtilis* cell growth and the use of NO_3_^−^ was better for enhancing surfactin synthesis in the presence of Mn^2+^. The results reported by Davis et al. supported our observations.[29] Under defined nutrition limited conditions, only 53.2 mg/L surfactin were obtained in cultures containing only ammonium, while in media containing only nitrate as the nitrogen source, poor growth was obtained (biomass concentration was 1.2–1.4 g/L). In cultures with both ammonium and nitrate, the surfactin concentration increased and reached a maximum when nitrate use occurred, causing the subsequent onset of nitrate-limited growth (surfactin concentration was 439 mg/L). One possible reason may be that due to the shorter assimilation pathway of ammonium, *B. subtilis* may prefer this nitrogen source, rather than nitrate, in order to support its own growth. However, the production of surfactin was enhanced when nitrate became the growth-limiting nutrient and the use of this nitrogen source may have been a switching signal to a secondary metabolism that promoted the synthesis of surfactin, as a secondary metabolite.[29,34]

To sum up, the addition of Mn^2+^ significantly changed the nitrogen metabolism of *B. subtilis*. The usage of NH_4_^+^ and NO_3_^−^ were both promoted at the same time. The NO_3_^−^ usage ability of *B. subtilis* was improved as the Mn^2+^ concentration increased, making nitrate the main nitrogen source instead of ammonium and a nitrate-limited growth, which led to the enhancement of the production of surfactin.

### Activities of enzymes in nitrogen assimilation in media with different concentrations of Mn^2+^

Based on the above analysis, nitrogen usage and surfactin synthesis by *B. subtilis* were closely related in the presence of Mn^2+^, which suggested that the enzymes involved in nitrogen assimilation require further study.

#### NR activity

**Figure 4.**
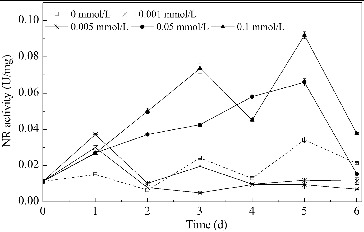
Changes in NR activity over time in media with different concentrations of Mn^2+^. Note: NR activity is expressed as micromoles of NADH oxidized per minute per milligram of protein.

**Figure 5.**
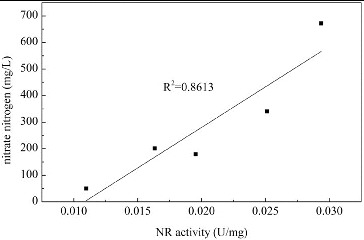
Correlation of NR activity and usage of nitrate nitrogen. Note: NR activity is expressed as micromoles of NADH oxidized per minute per milligram of protein.

The changes in NR activity over time in media with different concentrations of Mn^2+^ are shown in Figure 4. The NR activity of *B. subtilis* generally increased upon Mn^2+^ addition. In media with lower Mn^2+^ concentrations (0.001 and 0.005 mmol/L), the NR activity increased over the first two days, but then decreased to a relatively low level. In media with higher Mn^2+^ concentrations (0.05 and 0.1 mmol/L), the NR activity was significantly increased over the first five days. As shown in Figure 3, nitrate was mainly absorbed by *B. subtilis* over the first two days. Figure 5 shows that a significant positive correlation was observed between the nitrate usage and the NR activity over the first two days (*α* < 0.05). This indicates that the usage of nitrate is determined by the NR activity in media with Mn^2+^ as a promoting factor. This is because the step catalyzed by NR is the first and the speed-limiting step of the nitrate usage process.[32] It has also been reported that Mn^2+^ stimulated the activity of NR in *Clostridium perfringens* and with an addition of 0.1 mmol/L Mn^2+^, the NR activity improved by 58%.[43] For *B. subtilis* in this study, the addition of Mn^2+^ enhanced the NR activity, which directly improved the nitrate use and created a nitrate-limited condition, which could promote the production of surfactin.

#### GS and GOGAT activities

**Figure 6.**
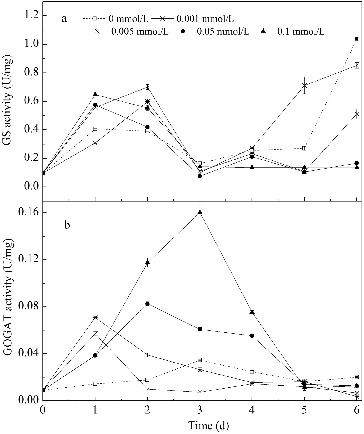
Changes in GS and GOGAT activities over time in media with different concentrations of Mn^2+^. Note: GS activity is expressed as absorption values increased per hour per milligram of protein. GOGAT activity is expressed as micromoles of NADH oxidized per minute per milligram of protein.

Changes in GS and GOGAT activities over time in media with different concentrations of Mn^2+^ are shown in Figure 6. In the first three days, GS activity first increased and then decreased during the cultivation. GS activity in the four media with Mn^2+^ was 50%–75% higher than that in the control medium, but the activities did not differ greatly from each other. This fits well with the observation that nitrogen usage in the four media with varying Mn^2+^ concentrations was similar in the different media, but higher than the control medium. In addition, the GS activity in media with higher Mn^2+^ concentrations (0.05 and 0.1 mmol/L) was at a low level after the third day, which coincided with the termination of nitrogen usage. The increase of the GS activity in the other three media after three days was unexpected, and the reason remained unclear. In general, the GS activity was promoted by Mn^2+^ addition but was not influenced by changes in Mn^2+^ concentration. Martinez-Espinosa et al. [44] also found that Mn^2+^ provoked an increase of the activity of the purified GS (purified from *Haloferax mediterranei*) at a concentration of 1 mmol/L due to its essential role as a cofactor.

As shown in Figure 6(b), Mn^2+^ had a stronger influence on GOGAT activity than on GS activity. The GOGAT activity was significantly higher in media with Mn^2+^ than in the control medium. The GOGAT activity was enhanced as the Mn^2+^ concentration increased. The highest activity was about 4.6 times more than that of the control medium on the third day at 0.1 mmol/L Mn^2+^. The GOGAT activity was maintained for a longer time in media with higher Mn^2+^ concentrations (0.05 and 0.1 mmol/L) than in media with lower Mn^2+^ concentrations (0.001 and 0.005 mmol/L). The GOGAT activity of *B. subtilis* was influenced by the ammonium content. The expression of *gltAB* encoding the GOGAT protein may be suppressed by the pleiotropic regulator *TnrA* in the absence of ammonium,[45] resulting in a lower cellular GOGAT activity. At lower Mn^2+^ concentrations (0.001 and 0.005 mmol/L), ammonium was used as the main nitrogen source and as the cultivation time increased, the ammonium decreased, finally reaching a concentration of 38 mg/L (see Figure 3). This may explain the decrease of GOGAT activity in media with lower Mn^2+^ concentrations (0.001 and 0.005 mmol/L).

On the whole, the presence of Mn^2+^ increased the activity of the nitrogen utilization and conversion enzyme system of *B. subtilis*. On one hand, the activity of NR was enhanced as the Mn^2+^ concentration increased, which also improved the use of nitrate and ammonium. The use of nitrate further promoted the synthesis of surfactin by increasing the secondary metabolism and led to a nitrate-limited growth, which was beneficial to the enhancement of surfactin production.[29] On the other hand, Mn^2+^ promoted nitrogen metabolism and transformation by increasing the GOGAT activity, which produced more free amino acids. The heptapeptide head group of surfactin is made up of seven amino acids,[10] thus an abundant free amino acid content is preferred by the surfactin synthesis process. It can be deduced that the presence of Mn^2+^ improved the synthesis of surfactin by influencing the nitrogen metabolism of *B. subtilis*.

## Conclusion

In this study, the role of Mn^2+^in promoting *B. subtilis*’ surfactin synthesis and substrate use was investigated from the perspective of metabolic enzymes’ activities. The addition of Mn^2+^ significantly improved the surfactin production by influencing the ammonium nitrate usage and the activity of the enzymes in nitrogen assimilation. This study showed an intimate relationship between nitrogen metabolism and surfactin biosynthesis in details, under the influence of Mn^2+^. It can be inferred that other convenient ways in industrial processes that can influence the nitrogen use of *B. subtilis* may also be effective in promoting surfactin yield, such as controlling the carbon nitrogen ratio and adding a moderate concentration of free amino acids in the medium.
